# Design of Soft Material Surfaces with Rationally Tuned Water Diffusivity

**DOI:** 10.1021/acscentsci.3c00208

**Published:** 2023-04-26

**Authors:** Audra J. DeStefano, My Nguyen, Glenn H. Fredrickson, Songi Han, Rachel A. Segalman

**Affiliations:** † Department of Chemical Engineering, University of California, Santa Barbara, California 93106, United States; ‡ Materials Research Laboratory, University of California, Santa Barbara, California 93106, United States; § Department of Materials, University of California, Santa Barbara, California 93106, United States; ∥ Department of Chemistry and Biochemistry, University of California, Santa Barbara, California 93106, United States

## Abstract

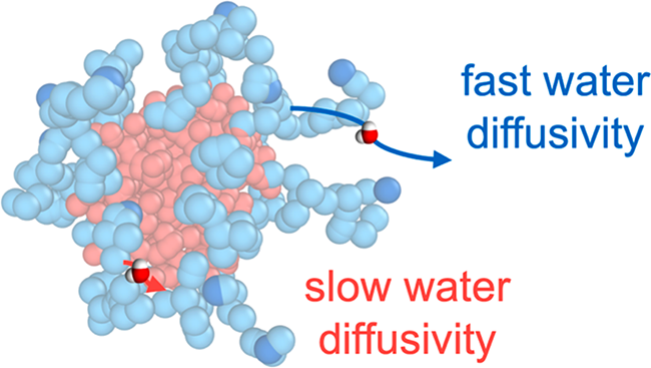

Water structure and dynamics can be key modulators of adsorption, separations, and reactions at soft material interfaces, but systematically tuning water environments in an aqueous, accessible, and functionalizable material platform has been elusive. This work leverages variations in excluded volume to control and measure water diffusivity as a function of position within polymeric micelles using Overhauser dynamic nuclear polarization spectroscopy. Specifically, a versatile materials platform consisting of sequence-defined polypeptoids simultaneously offers a route to controlling the functional group position and a unique opportunity to generate a water diffusivity gradient extending away from the polymer micelle core. These results demonstrate an avenue not only to rationally design the chemical and structural properties of polymer surfaces but also to design and tune the local water dynamics that, in turn, can adjust the local activity for solutes.

## Abstract 2

Sequence specificity enables multiple hydration environments to be accessed within a single material system. Local water diffusivity depends on the proximity to the core in polymeric micelles.

## Introduction

Equilibrium dynamics of hydration water mediate surface–solute interactions critical to sensing, catalysis, drug delivery, and advanced separations. Underlying water dynamics is the strong correlation between water diffusivity and local water structure as well as solvation thermodynamic properties. The development of next-generation functional materials for use in aqueous environments therefore necessitates the ability to design and tune surface water structure, dynamics, and thermodynamics.^1−4^ While some progress has been made in using surface chemistry and geometry to tune surface water properties,^4−8^ the engineering of versatile surfaces capable of producing multiple water environments, i.e., with different water volume fractions, diffusivity, and/or structure, that are readily accessible to solutes has not been achieved to date. This study demonstrates that water diffusivities can be made to vary as a function of radial distance in the fully hydrated corona of polymeric micelles, suggesting that incorporating functionalities at specific points within polymeric chains may enable us to access a range of user-defined water properties. The ability to access multiple water conditions within one material system will enable the precise engineering of specialized and even multifunctional waterborne materials.

Bioinspired materials, much like enzymes, can offer a route to producing local environments capable of facilitating interactions or reactions that require an environment that is distinct from that of bulk water. As one example, the amino acid l-proline catalyzes aldol reactions that form carbon–carbon bonds,^9^ but the l-proline-catalyzed aldol reaction is inactive in bulk water. Only when proline is incorporated on the surface of hydrophobic pockets formed by collapsed single-chain polymers can such reactions proceed in water.^10^ Critically, changing the chemistry of the hydrophobic monomers that line the folded hydrophobic pockets in which the reaction is carried out has been shown to tune the catalytic activity of l-proline while also decreasing the local water diffusivity.^10,11^ Water structuring has been proposed to stabilize transition states in aldol reactions, but studies of how the water environment of catalyst surfaces tunes broader classes of catalytic processes are only beginning.^10,12−15^ Again, what is missing is a material platform that allows access to multiple water environments, enabling a systematic investigation of water properties on catalytic processes. In this study, we focus on spatially and systematically tuning the local water density and diffusivity within the same soft material system.

Current theories suggest that the presence of a surface impacts water diffusivity by altering the governing hydrogen bond exchange mechanism in two ways.^16,17^ Bond exchange slows near interfaces due to (1) the steric hindrance of interacting with the surface and (2) the strength of hydrogen bonds between water and the surface.^16^ This suggests that the functional group position and chemistry on a surface or interface (or in this case within a polymer assembly) can tune the local water diffusivity. Discussions of the extent or size of the hydration shell and the degree to which water slows within it remain active.^18^ Differences in surface geometries, chemical compositions, and the sparsity of direct characterization techniques of hydration water make it highly challenging to generate a unified understanding of the spatial variation of water properties with respect to materials or molecular surfaces. It is generally agreed that water within the first few hydration layers (<1 nm) diffuses slower than bulk water, but few experiments offer the spatial resolution required to measure how far perturbations extend from a given surface.^19^ The determination and tuning of an experimental profile of water dynamics relative to a hydrated surface have therefore been elusive.

Here, polypeptoids serve as a class of model polymers in which the sequence of monomers and therefore the functional group or experimental probe position can be controlled.^20,21^ Indeed, Zhang and co-workers recently showed that the incorporation of charged groups at specific locations in polypeptoid amphiphiles allows for exquisite control over aqueous micelle size and structure.^22^ Our incorporation of nitroxide radical-based spin probes enables the use of Overhauser dynamic nuclear polarization (ODNP), a magnetic resonance technique uniquely capable of mapping translational water dynamics with ∼1 nm resolution. ODNP uses saturation of the electron paramagnetic resonance (EPR) signal of the spin probe to transfer polarization from the electron spin of the spin probe to nearby ^1^H nuclear spins of water and is described thoroughly elsewhere.^23,24^ Briefly, transferring electron spin polarization to nearby nuclei and subsequent exchange with bulk water enhance the ^1^H nuclear magnetic resonance (NMR) signal of water. Because the polarization transfer requires direct and close interaction between the electron and ^1^H nuclear spin and the transfer efficiency depends on the relative speed of movement of ^1^H with respect to the electron spin, the signal amplification effect is exclusively due to the dynamics of water within about 1 nm of a spin probe. Critically, site-directed spin labeling enables ODNP to characterize water diffusivity near specific regions of complex surfaces under ambient conditions. As a result, ODNP has resolved spatial heterogeneities in protein hydration and demonstrated that changes in surface chemistry drive differences in surface water behavior that impact protein and catalytic function.^1,11,25−27^ ODNP has also been successfully employed to map out the hydration profile across a peptide amphiphile (PA) fibril cross section, but the different water environments resolved in the study were focused on the water-depleted fibril interior and not the dynamic PA fibril surface accessible to interactions with various molecular constituents in solution.^28^

Measuring how water properties transition between the surface and bulk water, however, remains difficult even with ODNP because it is highly challenging to control the spatial location of the spin probe relative to the surface of interest. This study leverages the sequence-specificity of polypeptoids to control the spin probe position within micelles formed from amphiphilic polypeptoid chains and therefore enables measurements of water dynamics as a function of radial position within the micelle. Because the polypeptoid monomers are generally lacking in hydrogen bond donors (with the exception of a single monomer used to control micelle size), this model system allows hydrogen bond efficiency to be controlled by the choice of side chain and thereby simplifies polymer–water interactions to probe universal effects associated with polymer excluded volume.

## Results and Discussion

**Figure 1:**
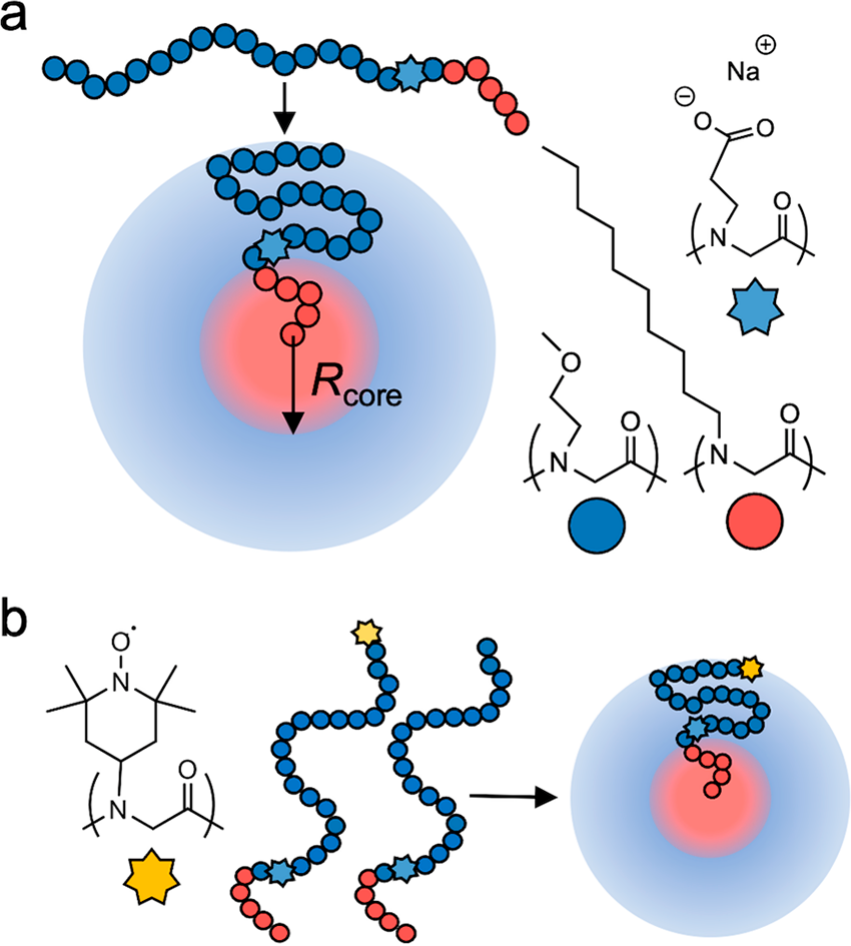
Sequence-defined polypeptoids enable spatial mapping of polymer and water properties. (a) Polypeptoids containing a hydrophobic 5-mer block and a hydrophilic 20-mer block self-assemble into spherical micelles. (b) Approximately one polypeptoid with a paramagnetic spin label is incorporated into each micelle. Precisely controlling the spin label position within the polypeptoid chain enables the characterization of average segmental motion and local water dynamics throughout the micelle.

**Figure 2:**
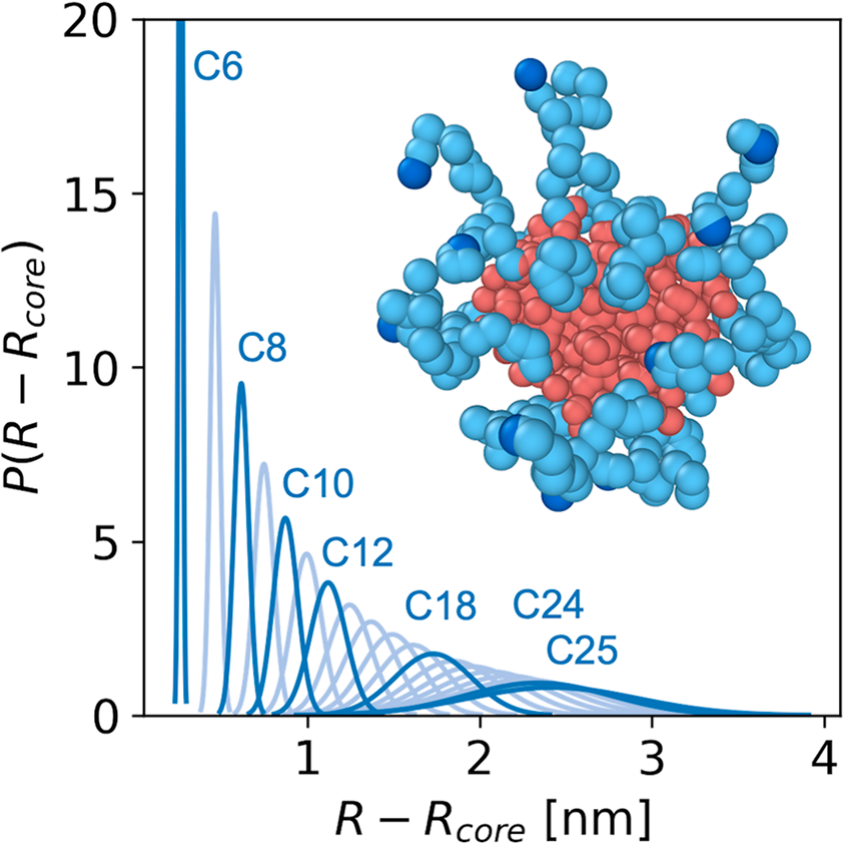
Distributions of the monomer position relative to the micelle core (*R* – *R*_core_) are determined by coarse-grained MD simulations. Distributions are plotted for each hydrophilic peptoid monomer, where C6 refers to the first hydrophilic monomer at the hydrophobic/hydrophilic transition and C25 represents the terminal hydrophilic monomer. The darker-blue distributions represent distinct monomer positions within the polypeptoid chains at which local water dynamics are measured by ODNP. Because C26 utilizes a spin label attached to the hydrated chain end, C25 is used as an approximate position.

In this study, the hydrophobic core of a polymeric micelle serves as a model surface surrounded by a water-rich corona. As shown in Figure 1a, spherical micelles are formed by amphiphilic polypeptoid chains containing hydrophobic and hydrophilic blocks with the hydrophobic block forming the dry core (pink in Figure 1) and the hydrophilic block spanning the hydrated corona (blue in Figure 1) of the micelle. The ether-like hydrophilic side chain is suitable for probing excluded volume effects because, despite containing one hydrogen bond acceptor, the side chain has shown impacts on water behavior very similar to those induced by nonpolar polypeptoids, suggesting inefficient hydrogen bonding.^3^ The size of the micelle (in water at an adjusted pH of 9) can be further adjusted via the position of a single charged peptoid monomer within the sequence (light-blue star in Figure 1) whose position is controlled by sequence-specific polypeptoid synthesis.^22^ In this work, the position of the charged peptoid monomer is held fixed to produce consistently sized micelles. To map water properties near the surface, we functionalize approximately one polypeptoid per micelle with a nitroxide spin label whose position is also precisely defined during synthesis (yellow star in Figure 1b, Section S2). The position of the spin-labeled monomer in terms of distance from the hydrophobic micelle core is determined by coarse-grained molecular dynamics (MD) simulations (Figure 2, Section S6). The local water diffusivity near the specific spin probe within the micelle is measured via ODNP (Section S5) to determine how water properties vary with the distance from the hydrophobic micelle core surface.

**Figure 3:**
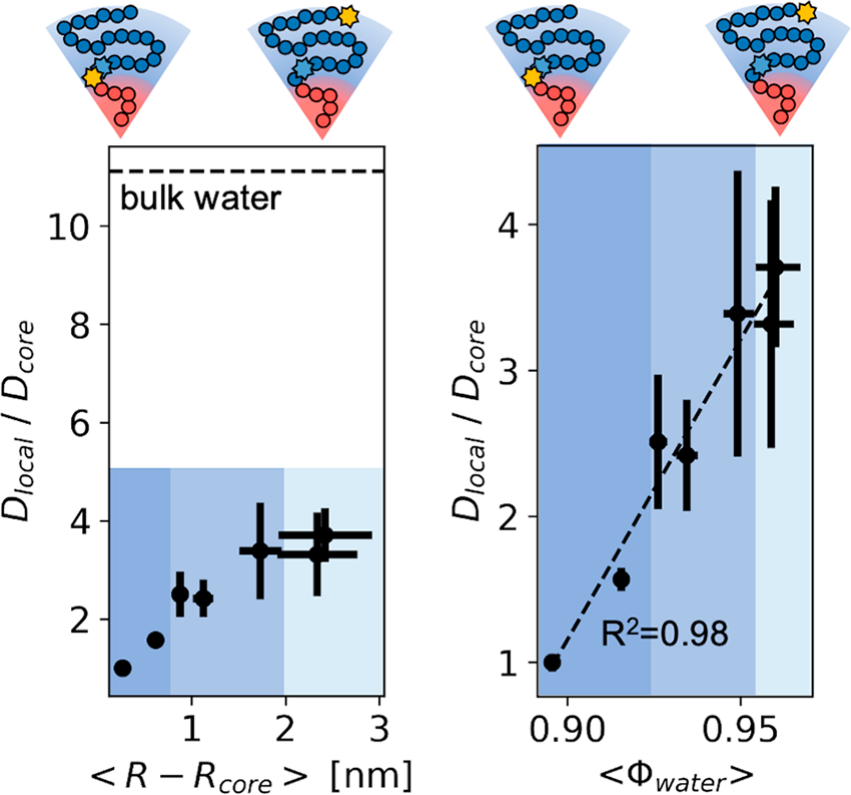
Water diffusivity is experimentally mapped throughout the micelle corona using seven distinct spin-label positions. *D*_local_ is slowest within about 1 nm of the hydrophobic surface and approaches a diffusivity about one-third that of bulk water toward the outside of the corona. Water diffusivity correlates more closely with the water volume fraction (⟨ϕ_water_⟩) within the corona than with the distance from the surface. This suggests that excluded volume predicts water behavior near nonpolar hydrophobic surfaces (the hydrophobic micelle core). The average distance from the core is calculated by coarse-grained MD simulations, and the average water volume fraction is calculated by ⟨ϕ_water_⟩ = 1 – *ϕ*_polymer_ (⟨*R*⟩). The diffusivity of bulk water is obtained from ref (23). Dark-blue shading represents water with buried character, while light-blue shading denotes surface-like behavior.

Combining knowledge of the position of the spin label within the micelle corona with sequence-controlled synthesis and ODNP experiments enables us to map out the water diffusivity gradient in the micelle corona. Specifically, local water diffusivities (*D*_local_) are measured at seven distinct monomer positions (Figure 2, Table S3). The measured local water diffusivities, *D*_local_, normalized by the measured water diffusivity near the core, *D*_core_, are plotted as a function of distance from the hydrophobic surface in Figure 3. The most dramatic retardation of water diffusivity occurs within about 1 nm of the hydrophobic micelle core surface. Beyond 1 nm but still within the corona, water translational diffusivities are observed to reduce to about one-third of that of bulk water. Figure 3 divides the corona into three regions (indicated in different shades of blue), based on ODNP hydration parameters from prior studies (Figure S4).^23^ This comparison suggests that water closest to the micelle core interacts strongly with the polymer chains, similar to buried water in polymers, proteins, and lipid vesicles. In contrast, water closest to the outside of the micelles shows surface-like water behavior, and water in between the two regimes exhibits intermediate dynamic characteristics. Previous studies also observed retarded water diffusivity near polymeric macromolecules and more extended soft surfaces,^1,3,11,29^ but spatially resolving water dynamics relied on the 3D structure of a protein scaffold and was never done as a function of distance from a surface within fully water-accessible volumes.

Because both the micelle core and corona consist almost entirely of monomers that do not efficiently hydrogen bond, universal polymer brush physics is assumed to describe water behavior. Figure 3 uses the volume fraction of water (ϕ_water_) as a proxy for the local environment because ϕ_water_ will increase as ϕ_polymer_ decreases with the distance from the micelle core. The radial distance from the center of the micelle core (*R*) relates to the polymer volume fraction (ϕ_polymer_) at a given point within the corona of charge-neutral block copolymer micelles by eq 1.^30−32^
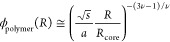
1

For polypeptoid micelles bearing a single ionic group per chain, Sternhagen et al. found that the model shown in eq 1 works well for describing the corresponding neutron scattering data. Here we employ eq 1 to relate the distance from the hydrophobic micelle surface within the corona to the water volume fraction, ϕ_water_(*R*) = 1 – ϕ_polymer_(*R*). The specific parameters selected are *R*_core_ = 1.8 nm (radius of the core) and *s* = 3.1 nm^2^ (interfacial area per polymer chain).^22^ The Flory exponent, ν, is equal to 3/5 under good solvent conditions, and the monomer size, *a*, is taken as 0.37 nm for polypeptoids in trans-amide conformations.^33^

When correlating the so-obtained water volume fraction, ϕ_water_, with the retardation of the ODNP-derived water diffusivity, *D*_local_*/D*_core_, we found a linear dependence, as shown in Figure 3, validating our hypothesis that the excluded volume inside the hydrated micelle corona closely correlates with local water diffusivity. Our finding is consistent with previous observations that protein hydration water diffusivity correlates with excluded volume near hydrophilic sites.^1^ This result suggests that enthalpic interactions between water and the polymer chains in polypeptoid micelles devoid of efficient hydrogen bond functionality are not the sole determinant of the water diffusivity gradient. Rather, steric effects play key roles in the inefficient hydrogen bonding systems studied here. Consequently, changing the geometry of polymer assemblies and surfaces, such as by moving the charged monomer location in the corona of our material system to make larger micelles, will likely alter the range and location of accessible water environments. We expect that the variation of polypeptoid chemistry to include specific interactions such as efficient hydrogen bonding or charged residues will impose further alterations in the water diffusivity landscape with respect to the micelle core surface. Nonetheless, our study demonstrates that proximity to a surface can be employed to systematically and significantly tune water diffusivity and thus the water environment within water-accessible locations in the micelle corona.

Because changes in water motion are often underpinned by variations in water structure,^19^ confinement may cause differences in water structuring that most likely underpin the reduced mobility of water near the hydrophobic surface. The structure of interfacial water balances packing forces with maximizing the number of hydrogen bonds and has been proposed to form “dangling bonds” in which one bonding group orients toward the hydrophobic surface rather than interacting with nearby water molecules.^34^ Because the polypeptoids used in this study do not hydrogen bond efficiently, the first hydration layer cannot form efficient hydrogen bonds with the surface and instead may reorient to form dangling bonds. Alternatively, water that does not efficiently bond to the polypeptoid surface may engage in stronger lateral interactions with neighboring water molecules, forming what has been introduced as a “wrap” water network in the recent literature.^35^ Differences in water orientation and packing influenced by interactions with nearby surfaces and chemical functionalities have been shown to consequently alter the water density and tetrahedrality that are descriptors of the water structural property.^18,19,36^ Indeed, molecular dynamics simulations predict increased water tetrahedrality with increasing hydrophobicity near single-chain polypeptoids, i.e., small-scale hydrophobic sites below 1 nm, that has been shown to correlate with slowed local water diffusivity.^3^ Furthermore, water with slowed local water diffusivity and increased tetrahedrality has also been shown to correlate with lower water entropy^3,19,37−40^ and hence can be exploited to tune solute–surface interaction.

**Figure 4:**
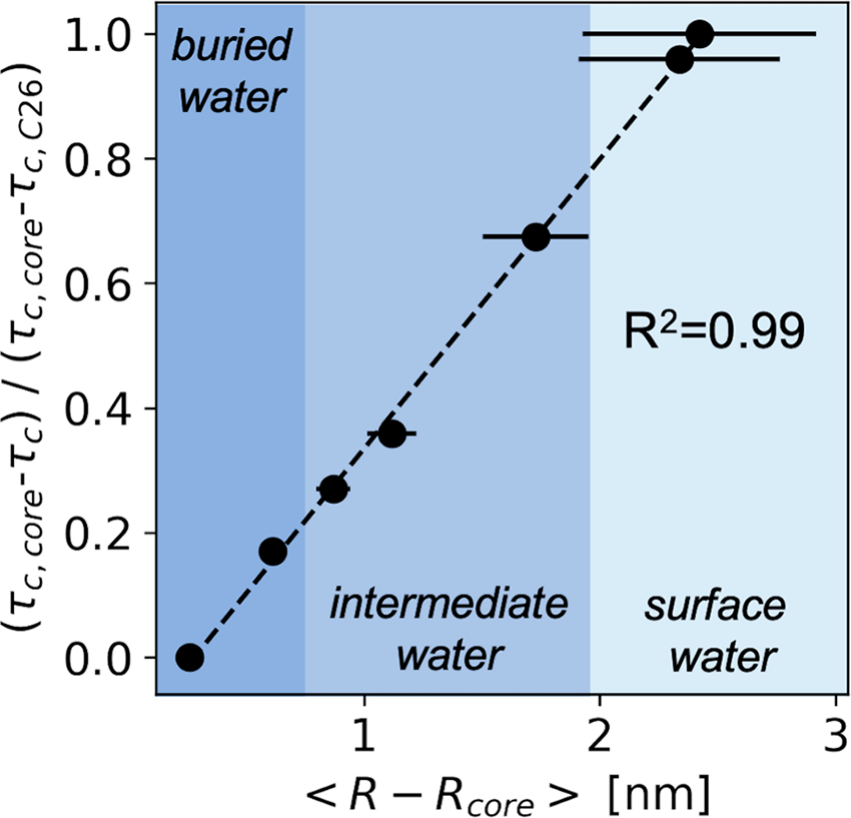
Spin label mobility serves as a proxy for the distance from the surface. Monomer distance from the micelle core (calculated by coarse-grained MD simulations) correlates linearly with the rotational correlation time (τ_c_) of EPR spin probes at seven spin label positions. Because spin probes are incorporated into the polymer backbone, changes in rotational correlation times are expected to reflect changes in segmental motion. Close to the micelle core, polymer chains are highly hindered (large τ_c_) due to dense packing in the core, while more water-rich regions experience higher mobility (short τ_c_). For each spin label position, τ_c_ is normalized to the range between the largest (edge of the core, τ_c,core_) and shortest (hydrophilic chain end, τ_c,C26_) τ_c_ values.

Understanding how water diffusivity varies spatially necessitates a knowledge of where each monomer, on average, is positioned. This study utilizes coarse-grained molecular dynamics simulations to estimate the distance from the core, while EPR may provide an experimental tool to approximate the relative spin label position. In addition to probing water properties with ODNP, spin labels enable polymer mobility mapping within assemblies by continuous wave EPR (cw-EPR).^41^ Lineshape analysis of cw-EPR spectra can be used to extract the rotational correlation time (τ_c_) of spin probes that corresponds to the time that the probe loses correlation to its initial orientation due to rotational motion.^42^ In the case of spin probes incorporated directly into the polypeptoid backbone, τ_c_ is expected to reflect the site-specific polymer segmental mobility.^43,44^ Normalized τ_c_’s plotted in Figure 4 demonstrate that mobility is most hindered closest to the micelle core, as expected. In fact, the monomer distance from the micelle core derived from MD simulation scales linearly with τ_c_ determined from cw-EPR lineshape analysis. Hence, cw-EPR provides a valuable experimental method to determine the relative position of the spin label from a surface within water-accessible volumes.

## Conclusions

In this study, we demonstrate that variations in excluded volume within a micellar corona can be utilized to tune the translational water diffusivity by up to a factor of 4 by combining sequence-defined polypeptoids capable of precisely defining the functional group position with ODNP. Local water diffusivity slows dramatically in the immediate vicinity of a hydrophobic surface but rapidly doubles within a distance of approximately 1 nm, likely due to increased free volume driving changes in water structure. This study showcases that controlling the proximity of functional groups to a surface offers a promising route to tuning material interactions with aqueous solutes for applications ranging from catalysis to water purification. Functional handles, such as proline, that can catalyze aldol condensation reactions can be readily incorporated into polypeptoids, making them an intriguing platform for probing the effect of local water environments on material performance, such as catalytic activity.

## Supporting Information Available

Methodological details, including synthetic protocols, polymer characterization via HPLC, MALDI, DLS, cw-EPR spectra, ODNP hydration parameters, and MD simulation parameters (PDF)
