# Supplementary material for: Design of Soft Material Surfaces with Rationally Tuned Water Diffusivity
Source: ACS Cent Sci. 2023 Apr 26;9(5):1019–24. doi: 10.1021/acscentsci.3c00208 (PMC10214527; doi:10.1021/acscentsci.3c00208)
Supplement: Supplementary file 1 — oc3c00208_si_001.pdf [file oc3c00208_si_001.pdf]

Supplementary Materials for

Design of soft material surfaces with rationally tuned water diffusivity

Audra J. DeStefano[1], My Nguyen,[1] Glenn H. Fredrickson,[1,2,3] Songi Han[1,4*], Rachel A. Segalman[1,2,3,4]*

[1]Department of Chemical Engineering, University of California, Santa Barbara, California 93106 U.S.A.

[2]Materials Research Laboratory, University of California, Santa Barbara, California, 93106 U.S.A.

[3]Department of Materials, University of California, Santa Barbara, California, 93106 U.S.A.

[4]Department of Chemistry and Biochemistry, University of California, Santa Barbara, California, 93106 U.S.A.

*Corresponding authors: songi@chem.ucsb.edu, segalman@engineering.ucsb.edu

Contents

1. Safety 3

2. Polypeptoid Synthesis 4

3. Micelle Assembly 7

4. Electron Paramagnetic Resonance (EPR) 8

5. Overhauser Dynamic Nuclear Polarization (ODNP) 9

6. Coarse-grained molecular dynamics (CG MD) 11

7. References 14

1. Safety

Polypeptoid synthesis makes use of flammable, carcinogenic, toxic, and sensitizing chemicals. As such, all synthetic steps are conducted within a fume hood or under a snorkel using appropriate eye protection, lab coats, and tri-layer gloves.

2. Polypeptoid Synthesis

Micelle-forming polypeptoids with precisely placed nitroxide spin labels are synthesized using established methods on an automated Prelude peptide synthesizer.[1] All polypeptoids are grown on a rink-amide resin (loading 0.62 mmol/g, 100 μmol scale). The resin is deprotected with 20% 4-methylpiperdine in dimethylformamide (DMF). Each monomer addition is then divided into two steps separated by DMF washes. All steps are performed at room temperature. The first consists of bromoacetylation for 20 minutes with 1.2 M bromoacetic acid and 0.4 M N,N'-diisopropylcarbodiimide in DMF. The second imparts monomer functionality through incorporation of a primary amine (1M in DMF) for 2 hours. A hydrophobic block of 5 monomers is first synthesized using N-decylamine (Ndc) followed by one monomer from N-methoxyethylamine (Nme) and one from β-Alanine tert-butyl ester (Nce). Finally, 18 monomers are incorporated from N-methyoxyethylamine. For each spin labeled sequence, a solution of 4-amino-2,2,6,6-tetramethylpiperidinyloxyl (Ntmp) is used rather than N-methoxyethylamine at the specified position with monomer one being the initial hydrophobic group. For example, sequence C6 contains a spin label at position 6 rather than a methoxyethyl sidechain at the hydrophobic-hydrophilic interface. Upon completion of the polypeptoid sequence, the polypeptoids are acetylated for 30 minutes in 0.4 M pyridine and 0.4 M acetic anhydride in DMF. We note that formylation would likely be preferable to acetylation to prevent end group cleavage if it does not impact polypeptoid self-assembly.[1-3] β-Alanine tert-butyl ester is purchased as a hydrochloride and extracted from ethyl acetate and basic water. All other chemicals are used as received.

A cocktail of trifluoroacetic acid : water : triisopropylsilane (95 : 2.5 : 2.5, v/v/v) is used to cleave polypeptoids from the solid support. After immersion in the cocktail for 2 hours, the resin is filtered and washed with dichloromethane. The collected solution is dried under vacuum and lyophilized from acetonitrile and water (1 : 1, v/v). Because the cleavage process causes spin labels to disproportionate, polypeptoids are stirred in a 7 N ammonia in methanol : water (9 : 1, v/v) solution for 4-12 hours. The solution is then removed by vacuum and the samples are lyophilized from acetonitrile and water (1 : 1, v/v). This method does not regenerate all radicals but enables sufficient labeling for EPR and ODNP experiments.

To confirm the presence of the target compounds, polypeptoid samples are characterized with matrix-assisted laser desorption/ionization (MALDI) spectrometry and high-pressure liquid chromatography (HPLC). MALDI is done on a Bruker Microflex LRF MALDI TOF mass

spectrometer. Alpha-cyano matrix is prepared in tetrahydrofuran. Matrix-sample mixtures are spotted onto a polished steel MALDI target plate. Mass spectra are collected in positive reflectron mode. HPLC is done on a Waters Acquity H-class Ultra High Pressure Liquid Chromatography system. All samples are dissolved in acetonitrile and water (1 : 1, v/v) with 0.1% formic acid and separated using a 50-100% acetonitrile gradient. The detected wavelength is 214 nm.

Table S1. Polypeptoid sequences and molecular weights.

Sample	Sequence	Calc (m/z)	Found (m/z)	Ion type
Unlabeled	$Nme_{18}NceNmeNdc_5$	3362.24	3363.6	$[M+H]^+$
C6	$Nme_{18}NceNtmpNdc_5$	3457.38	3482.6	$[M+Na]^+$
C8	$Nme_{17}NtmpNceNmeNdc_5$	3458.48	3481.1	$[M+Na]^+$
C10	$Nme_{15}NtmpNme_2NceNmeNdc_5$	3458.48	3481.2	$[M+Na]^+$
C12	$Nme_{13}NtmpNme_4NceNmeNdc_5$	3458.48	3482.3	$[M+Na]^+$
C18	$Nme_7NtmpNme_{10}NceNmeNdc_5$	3457.38	3482.0	$[M+Na]^+$
C24	$Nme_1NtmpNme_{16}NceNmeNdc_5$	3457.38	3481.2	$[M+Na]^+$
C26	$NtmpNme_{18}NceNmeNdc_5$	3572.53	3597.3	$[M+Na]^+$

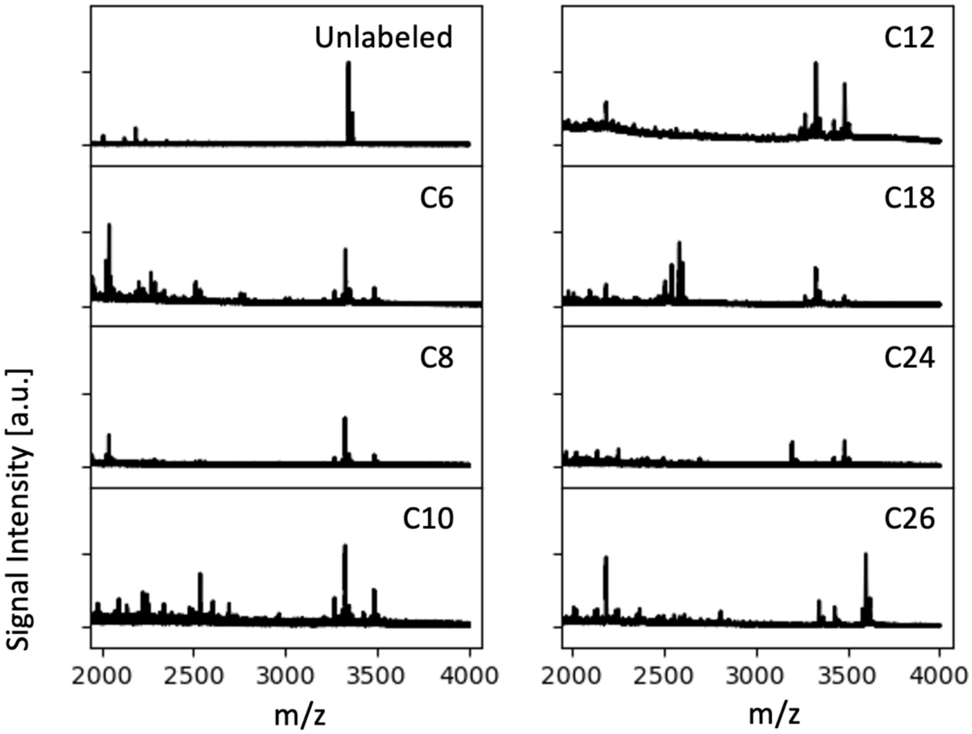

Figure S1. MALDI confirms presence of desired product. The dominant biproduct for most samples is a sequence in which the terminal monomer is removed during the peptoid cleavage process.

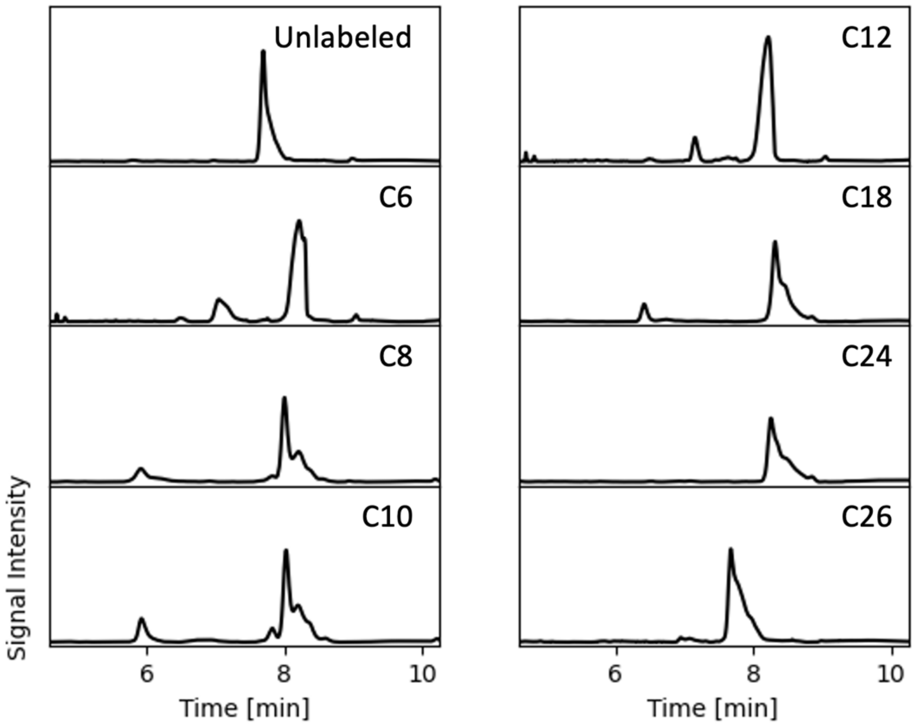

Figure S2. HPLC traces for labeled and unlabeled polypeptoids.

3. Micelle Assembly

Micelles are prepared by suspending 5 mg/mL of polypeptoid in filtered, ultrapure water following the method described by Sternhagen et al.[4] Fewer than one spin labeled chain, on average, is incorporated into each micelle to target a spin concentration of 100-200 μM. The pH is adjusted to 9 using sodium hydroxide.

Hydrodynamic radii are measured with light scattering to confirm that spin label incorporation does not change micelle size. All measurements are conducted at 20 °C at a concentration of 5 mg/mL using a Brookhaven Instruments BI-200SM goniometer with a scattering angle of 90°. The system utilizes a 500 mW dye-pumped solid state laser that operates at 532 nm. The R_h measured here are larger than those reported by Sternhagen et al.[4] This is likely due to differences in filtering protocols during sample preparation or the presence of small numbers of worm-like micelles (also reported by Sternhagen et al).

Table S2. Hydrodynamic radii obtained via light scattering show that incorporation of a small number of spin labeled chains does not change the micelle size.

**Sample**	R_h **[nm]**
Unlabeled	33 ± 3
C6	30 ± 5

4. Electron Paramagnetic Resonance (EPR)

Spin label concentrations and spin label mobilities are measured via cw-EPR on micelle solutions prepared at a concentration of 5 mg/mL in water with spin concentrations of 100-200 μM. A quartz round capillary tube of 0.60 mm inner diameter and 0.84 mm outer diameter is loaded with 3.5 μL of solution and sealed at one end with beeswax and at the other with Critoseal. The dispersive electron paramagnetic resonance (EPR) spectrum is obtained with a fixed frequency (9.8 GHz) at 20 dB, while the magnetic field is swept with a modulation frequency of 140.0 kHz and a modulation amplitude of 0.70 G. Spin concentrations were obtained by double integration of the spectrum. Lineshape analysis is performed using the Multicomponent software[5] to obtain rotational correlation times (τ_c) for each sample. Cw-EPR spectra and fits are shown in Figure S3.

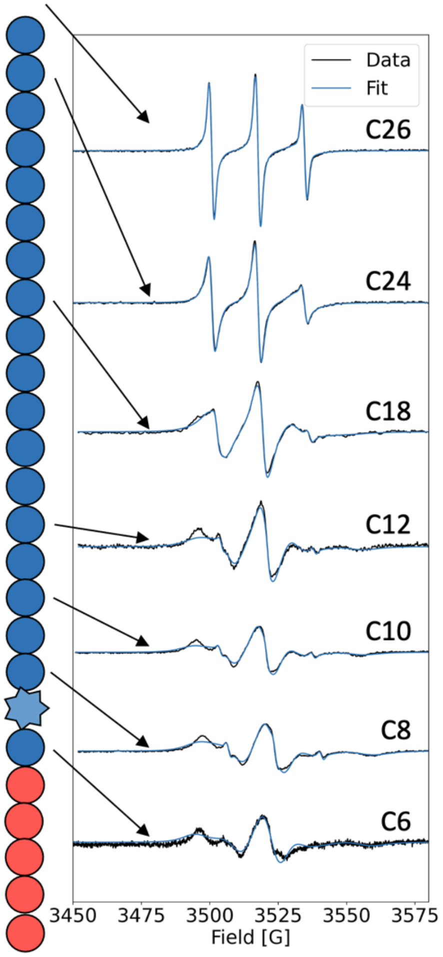

Figure S3. Raw cw-EPR spectra (black) and their corresponding fits (blue) for seven spin label positions within the polymeric micelles.

5. Overhauser Dynamic Nuclear Polarization (ODNP)

ODNP experiments utilize the samples prepared for EPR in section S4. Sample temperature is maintained at 18°C in a ER4123D dielectric resonator using a stream of compressed air. ODNP is performed at 0.35 T at a 14.8 MHz ^{1}H Larmor frequency and at 9.8 GHz electron spin Larmor frequency using a home-built U-shaped NMR coil. An inversion-recovery pulse sequence acquires proton spin-lattice relaxation times (T_1). Following the protocol described by Franck et al.,[6] $T_{1,0,0}$ is determined to be 2.37 for unlabeled micelles. Hydration parameters ($T_{1,0}$, $T_{1,0,0}$, k_σ, coupling factor (ξ), the translational correlation time of water interacting with the electron spin by dipolar cross-relaxation (τ_{corr}), and D_{local}) are calculated from ODNP experiments using previously established methods implemented through a Python-based software package called dnpLab.[7] In brief, the Overhauser effect causes an enhancement in NMR signal by saturation of the EPR signal by microwave irradiation. The electron-nuclear spin cross-relaxation rate, k_σ, is extracted from this saturation and combined with $T_{1,0,0}$ to determine the self-relaxation rate (k_ρ). Dividing k_σ by k_ρ yields ξ. ξ is combined with the analytical form of the spectral density function using the force free hard sphere model to calculate τ_{corr}. Finally, the local water diffusivity within 1 nm of the spin probe is calculated using equation 1 where $\tau_{\mathrm{corr,bulk}}$ is τ_{corr} for bulk water, $D_{\mathrm{H_2O}}$ is the diffusivity of water, and D_{SL} is the diffusivity of the spin label.

$$D_{\mathrm{local}} \equiv \frac{\tau_{\mathrm{corr,bulk}}}{\tau_{\mathrm{corr}}}(D_{\mathrm{H_2O}} + D_{\mathrm{SL}}) \qquad \text{(Eqn. 1)}$$

Hydration parameters ($T_{1,0}$, ξ, and D_{local}) are listed in Table S3 for each sample. The environment of water can be estimated by τ_{corr} and ξ (Figure S4).

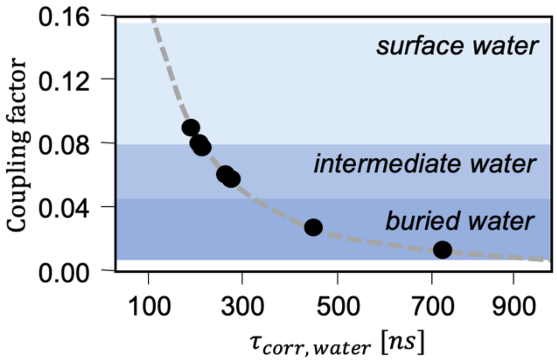

Figure S4. The buried, intermediate, or surface-like character of water is classified by the coupling factor (ξ) and the translational correlation time of water interacting with the electron spin by dipolar cross-relaxation (τ_{corr}). The dashed line represents the relationship between ξ and τ_{corr} as connected through the force-free hard sphere model at a field strength of 0.35 T (reproduced from ref 6).

Table S3. Hydration parameters ($T_{1,0}$, ξ, and D_{local}) obtained via ODNP for micelle samples with varied spin probe position. Standard deviations are calculated based on three experimental trials.

**Sample**	$\boldsymbol{T_{1,0}}$	$\boldsymbol{\xi}$	$\boldsymbol{D_{local}}$ **[10^{10} m^2/s]**
C6	1.77 ± 0.01	0.014 ± 0.001	2.05 ± 0.09
C8	1.26 ± 0.01	0.028 ± 0.001	3.22 ± 0.06
C10	1.67 ± 0.09	0.056 ± 0.014	5.15 ± 0.91
C12	1.92 ± 0.06	0.053 ± 0.011	4.98 ± 0.75
C18	1.93 ± 0.13	0.083 ± 0.029	6.96 ± 1.98
C24	1.98 ± 0.08	0.081 ± 0.026	6.81 ± 1.71
C26	2.03 ± 0.03	0.093 ± 0.016	7.61 ± 1.08

6. Coarse-grained molecular dynamics (CG MD)

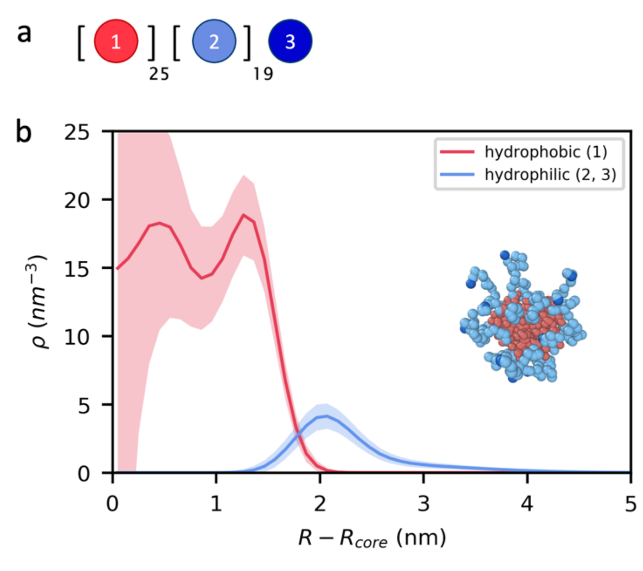

Figure S5. (a) Schematic of the polymer chain consisting of a 25-mer block of the hydrophobic bead species 1, a 19-mer block of the internal hydrophilic bead species 2, and one terminal hydrophilic bead species 3. (b) Density profiles of the hydrophobic and hydrophilic beads as a function of the distance from the micelle center from CG MD.

Amphiphilic polypeptoid chains are modeled as bead-spring polymers consisting of a 25-mer block of the hydrophobic bead species 1, a 19-mer block of the internal hydrophilic bead species 2, and one terminal hydrophilic bead species 3 (Figure S5a). The number of hydrophobic beads is selected to reproduce the size of the experimental hydrophobic core, while the number of hydrophilic beads matches the number of hydrophilic polypeptoid monomers. Each solvent molecule is modeled explicitly as one single CG bead of type w that resembles water. The coarse-grained (CG) force field comprises of harmonic bond potentials of the form

$$\beta U_{b,\alpha\gamma}(r) = \frac{k_{\alpha\gamma}}{2} r^2$$

where $\beta = 1/k_B T$ and $k_{\alpha\gamma}$ is the spring constant for a bond between bead species α and γ. The excluded volume between all site pairs, including bonded pairs, is described by the non-bonded pairwise repulsive Gaussian potential

$$\beta U_{ev,\alpha\gamma}(r) = v_{\alpha\gamma} e^{-r^2/2(a_\alpha^2 + a_\gamma^2)}$$

where $v_{\alpha\gamma}$ describes the excluded volume strength between bead species α and γ and a_α is the interaction range of CG-site α. We use the interaction range $a_\alpha \sim \rho_w^{1/3}$, where ρ_w is the water density measured in an atomistic simulation at 298.15 K and 1 atm. v_{ww} is chosen to approximately reproduce water's compressibility ($\approx 4.51 \times 10^{-10}$ Pa^{-1}); this determines the CG pressure $P = 283.95\ k_B T/nm^3$. The remaining excluded volume parameters $v_{\alpha\gamma}$ are determined to capture the increasing hydrophobicity of the monomers: terminal hydrophilic species 3 < internal hydrophilic species 2 < hydrophobic species 1. Spring constants $k_{\alpha\gamma}$ are chosen such that equilibrium bond lengths are around 0.3 nm, approximately the monomer size in the experiment. This choice of parameters results in the micelle structure where the core is made up of species 1 and the shell consists of species 2 and 3 (Figure S5b). The CG forcefield parameters are tabulated and provided in Table S4.

The micelle simulation consists of 13 polymer chains and 134325 solvent molecules in a cubic box and is conducted with the OpenMM simulation package.[8] A 2.22 nm cutoff for the non-bonded Gaussian interactions and a time step of $dt = 0.02\ \tau$ are used. The initial configuration is relaxed for 20 τ and the trajectory is collected for analysis in the last 20,000 τ. The temperature is set to $T = 298.15$ K using the Langevin thermostat with a relaxation time of 100 dt, while the pressure is set to P using the Monte Carlo isotropic barostat with the update frequency of 25 dt. The average box side length from the production run is $\sim$ 16 nm. As evidenced by the density profile in Figure S5b, the resulting micelle has core and corona sizes comparable to those determined experimentally ($R_c = 1.8$ nm, $R_m = 4.47$).

Table S4. Coarse-grained parameters

**Parameter**	**Value**	**Unit**
$\boldsymbol{k_{11}}$	32.986	$k_B T$/nm^2
$\boldsymbol{k_{12}}$	49.345	$k_B T$/nm^2
$\boldsymbol{k_{22}}$	29.612	$k_B T$/nm^2
$\boldsymbol{k_{23}}$	43.323	$k_B T$/nm^2
$\boldsymbol{a_1}$	0.311	nm
$\boldsymbol{a_2}$	0.375	nm
$\boldsymbol{a_3}$	0.311	nm
$\boldsymbol{a_w}$	0.311	nm
$\boldsymbol{v_{11}}$	1.680	$k_B T$
$\boldsymbol{v_{12}}$	1.260	$k_B T$
$\boldsymbol{v_{13}}$	1.028	$k_B T$
$\boldsymbol{v_{1w}}$	0.953	$k_B T$
$\boldsymbol{v_{22}}$	1.078	$k_B T$
$\boldsymbol{v_{23}}$	0.957	$k_B T$
$\boldsymbol{v_{2w}}$	0.538	$k_B T$
$\boldsymbol{v_{33}}$	0.0	$k_B T$
$\boldsymbol{v_{3w}}$	0.473	$k_B T$
$\boldsymbol{v_{ww}}$	0.339	$k_B T$

7. References

(1) Connolly, M. D.; Xuan, S. T.; Molchanova, N.; Zuckermann, R. N. Submonomer synthesis of sequence defined peptoids with diverse side-chains. *Method Enzymol* **2021**, *656*, 241-270. DOI: 10.1016/bs.mie.2021.04.022.

(2) Kim, S.; Biswas, G.; Park, S.; Kim, A.; Park, H.; Park, E.; Kim, J.; Kwon, Y. U. Unusual truncation of N-acylated peptoids under acidic conditions. *Org Biomol Chem* **2014**, *12* (28), 5222-5226. DOI: 10.1039/c3ob42572j.

(3) Wijaya, A. W.; Nguyen, A. I.; Roe, L. T.; Butterfoss, G. L.; Spencer, R. K.; Li, N. K.; Zuckermann, R. N. Cooperative Intramolecular Hydrogen Bonding Strongly Enforces cis-Peptoid Folding. *J Am Chem Soc* **2019**, *141* (49), 19436-19447. DOI: 10.1021/jacs.9b10497.

(4) Sternhagen, G. L.; Gupta, S.; Zhang, Y. H.; John, V.; Schneider, G. J.; Zhang, D. H. Solution Self-Assemblies of Sequence-Defined Ionic Peptoid Block Copolymers. *J Am Chem Soc* **2018**, *140* (11), 4100-4109. DOI: 10.1021/jacs.8b00461.

(5) *Multicomponent*; 2021. https://sites.google.com/site/altenbach/labview-programs/epr-programs/multicomponent (accessed January 15, 2022).

(6) Franck, J. M.; Pavlova, A.; Scott, J. A.; Han, S. Quantitative cw Overhauser effect dynamic nuclear polarization for the analysis of local water dynamics. *Prog Nucl Mag Res Sp* **2013**, *74*, 33-56. DOI: 10.1016/j.pnmrs.2013.06.001.

(7) *DNPLab*; 2021. http://dnplab.net/ (accessed February 4, 2021).

(8) Eastman, P.; Swails, J.; Chodera, J. D.; McGibbon, R. T.; Zhao, Y. T.; Beauchamp, K. A.; Wang, L. P.; Simmonett, A. C.; Harrigan, M. P.; Stern, C. D.; et al. OpenMM 7: Rapid development of high performance algorithms for molecular dynamics. *Plos Comput Biol* **2017**, *13* (7). DOI: ARTN e1005659
10.1371/journal.pcbi.1005659.
